# Adaptive phenotypic response to climate enabled by epigenetics in a K-strategy species, the fish *Leucoraja ocellata* (Rajidae)

**DOI:** 10.1098/rsos.160299

**Published:** 2016-10-26

**Authors:** Jackie Lighten, Danny Incarnato, Ben J. Ward, Cock van Oosterhout, Ian Bradbury, Mark Hanson, Paul Bentzen

**Affiliations:** 1 School of Environmental Sciences, University of East Anglia, Norwich Research Park, Norwich NR4 7TJ, UK; 2 Dipartimento di Scienze della Vita e Biologia dei Sistemi, Università di Torino, Via Accademia Albertina 13, 10123 Torino, Italy; 3 Human Genetics Foundation (HuGeF), via Nizza 52, 10126 Torino, Italy; 4 Earlham Institute, Norwich Research Park, Norwich NR4 7UG, UK; 5 Department of Fisheries and Oceans, 80 White Hills Road, St John's, Newfoundland, CanadaA1C 5X1; 6 Department of Fisheries and Oceans, Gulf Region, 343 Université Avenue, Moncton, New Brunswick, CanadaE1C 9B6; 7 Marine Gene Probe Laboratory, Department of Biology, Dalhousie University, Halifax, Nova Scotia, CanadaB3H 4R2

**Keywords:** epigenetics, climate change, skate, fish, phenotypic adaptation, K-strategy

## Abstract

The relative importance of genetic versus epigenetic changes in adaptive evolution is a hotly debated topic, with studies showing that some species appear to be able to adapt rapidly without significant genetic change. Epigenetic mechanisms may be particularly important for the evolutionary potential of species with long maturation times and low reproductive potential (‘K-strategists’), particularly when faced with rapidly changing environmental conditions. Here we study the transcriptome of two populations of the winter skate (*Leucoraja ocellata*), a typical ‘K-strategist’, in Atlantic Canada; an endemic population in the southern Gulf of St Lawrence and a large population on the Scotian Shelf. The endemic population has been able to adapt to a 10°C higher water temperature over short evolutionary time (7000 years), dramatically reducing its body size (by 45%) significantly below the minimum maturation size of Scotian Shelf and other populations of winter skate, as well as exhibiting other adaptations in life history and physiology. We demonstrate that the adaptive response to selection has an epigenetic basis, cataloguing 3653 changes in gene expression that may have enabled this species to rapidly respond to the novel environment. We argue that the epigenetic augmentation of species evolutionary potential (its regulation though gene expression) can enable K-strategists to survive and adapt to different environments, and this mechanism may be particularly important for the persistence of sharks, skates and rays in the light of future climate change.

## 1. Introduction

Throughout evolutionary history, changes in global temperature have affected the survival and distribution of species [1–3]. In particular, ocean temperature is an important environmental factor governing patterns of biodiversity in marine ecosystems though its effects on productivity [4] and ecophysiology [5,6]. Indeed, rising water temperatures can affect fishes by reducing the efficiency of aerobic respiration, as metabolic rate (and so oxygen demand) increases in warmer waters [7,8]. This issue is compounded further by the fact that the concentration of dissolved oxygen decreases as water temperature increases. As ocean temperatures have risen, the mean latitude of some species ranges has started to shift towards the poles in response to changing conditions in their native habitat [9–11]. This enables species to escape the effects of rapid climate change, which currently is significantly accelerated beyond that of natural climatic cycles [12]. However, it is predicted that species may adapt to rising temperatures though physiological trade-offs to cope with poorer oxygen metabolism. Adaptation to lower oxygen concentrations alongside increased metabolic demand is likely to cause reductions in individual body size, fecundity, and species abundances [13]. Even though contemporary climate change has been accelerated through increased carbon emissions, natural episodes of temperature change have still occurred relatively quickly across geological timescales [12]. A wealth of fossil and genetic evidence suggests such rapid climate shifts provided little to no opportunity for both terrestrial and marine organisms to evolve in response to environmental changes, and thus many species either became extinct or populations were reduced to restricted areas of suitable habitat during such periods [1]. Also in the present climate change scenario, local species extinctions are predicted to hit the subpolar regions, whereas significant species invasions in the Arctic are projected to result in dramatic species turnovers [14,15].

Traditional evolutionary genetic theory developed by Fisher [16] states that the rate of increase in fitness of any organism at any time is equal to its genetic variance in fitness at that time. This implies that a species with less genetic variation may have less adaptive potential under rapidly changing environmental conditions compared with a species that has high genetic variation. The recent surge in studies on epigenetics shows, however, that species can undergo rapid change through regulation of gene expression driving phenotypic adaptation in response to new environmental conditions. Importantly, this variation in gene expression may manifest in a population very quickly as the epigenetic profile can be inherited between generations in some, but not all, organisms [17–19]. Environmental stress may cause rapid epigenetic changes, and if some of these changes can escape epigenetic reprogramming in the germ line, expression variation may perpetuate in a form of Lamarckian inheritance. Epigenetic changes may therefore provide the precursory adaptations to facilitate a response to natural selection, potentially resulting in speciation over longer timescales. However, even without the inheritance of an epigenetic marker, evolutionary responses may be contingent on epigenetic phenotypic plasticity within each generation. For example, in marine microalgae, more plastic genotypes tend to show a greater evolutionary potential on average [20]. Indeed, in order to adapt to a novel environment at the DNA level, a species first needs to be able to tolerate and persist in that environment, and epigenetic-driven phenotypic plasticity (or adaptation though regulation of gene expression) may provide genotypes with a physiological buffer to withstand the environmental changes.

Interestingly, recent evidence has shown that marine fishes can very quickly switch genes on or off in response to warming waters [21]. Experiments showed that over the space of just two generations, the damselfish (*Acanthochromis polyacanthus*) exhibited transgenerational accumulation of differentially expressed genes involved in thermal adaptation of aerobic capacity, as well as genes involved in immune and stress responses. These epigenetic changes fit with predictions of how inherited gene expression profiles may allow subsequent generations to better deal with rising temperatures. Experimental studies using wild-caught guinea pigs have even shown that inheritance of epigenetic modifications may be important in determining the survival of mammalian species during increased environmental warming [22]. To date, however, no evidence has been reported that suggests climate change (or even range wide differences in climate) has led to morphological and ecophysiological adaptation in natural fish populations through regulation of gene expression.

**Figure 1.**
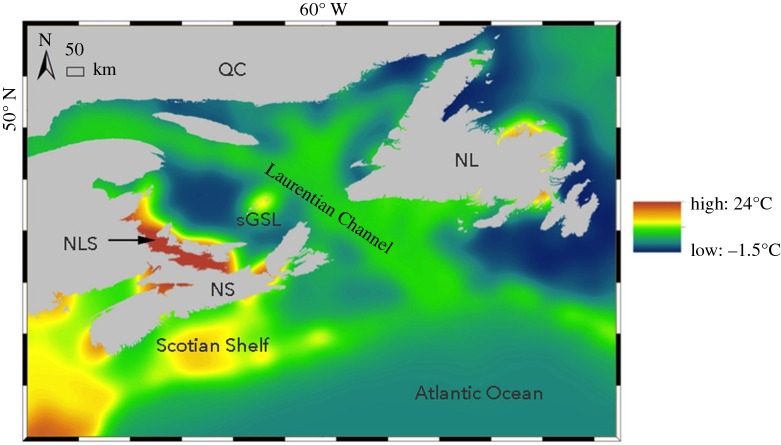
Map shows variation in mean summer bottom temperatures of oceanic waters in Atlantic Canada (Nova Scotia, NS; Québec, QC; Newfoundland, NL). The southern Gulf of St Lawrence (sGSL) population of winter skate (*Leucoraja ocellata*) is concentrated in the Northumberland Strait (NLS) during summer, when samples were collected here, and when this shallow area is ice-scoured, moves to deeper water towards the Laurentian Channel, which still experiences near freezing temperatures (−1.7°) (26). The Scotian Shelf population lives in a similar oceanic area where both the winter temperatures are milder and summer temperatures cooler. The summer bottom temperatures vary between these regions by *ca* 10°C. Although some warmer shallow waters are also found in the Bay of Fundy (NS), skates tend to move in and out of shallow regions into cooler deeper regions during the summer, compared with the NLS, which is surrounded by much deeper and colder water.

By studying individuals from populations of the winter skate (Chondrichthyes, Elasmobranchii, *Leucoraja ocellata*) in Atlantic Canada (figure 1), we investigate the hypothesis that wild fish populations can develop substantial morphological and ecophysiological variation as a consequence of differential gene expression between different climatic conditions. The effect of temperature on skate biology is poorly understood; however, from the few studies available, temperature may be important in determining the timing of growth patterns [23] and variation in reproductive life-history traits [24]. Previous studies have shown that winter skate in the southern Gulf of St Lawrence (sGSL) population appear to be an undescribed endemic, which are substantially divergent from the Scotian Shelf population in morphology (e.g. significantly smaller body size despite no significant difference in growth rate), life history (e.g. shorter life span) and ecophysiology (e.g. inhabit significantly warmer waters; table 1) [25,26]. However, the two populations are phylogenetically indistinguishable using the standard DNA barcoding gene, cytochrome oxidase I [27]. Worryingly, winter skate is listed as an endangered species by the IUCN, with the sGSL population on the brink of extirpation [28]. Skate numbers are particularly vulnerable to high rates of fisheries bycatch [29] or overexploitation by invading predators (grey seals, *Halichoerus grypus*, in Atlantic Canada, [28,30]) owing to their long maturation times and relatively low fecundity, which they share in common with other elasmobranchs. These life-history traits are characteristic for a K-strategist, but it also means that elasmobranchs have a low propensity for rapid evolutionary change [31,32]. Paradoxically, however, skates are noted for having unusually high levels of biodiversity and endemism around the globe, collectively spanning vast regions that vary markedly in ecological conditions [33,34]. For such K-strategy species with a modest evolutionary potential, epigenetic-driven phenotypic adaptation may be of particular importance to provide a physiological buffer to withstand the environmental changes. Interestingly, the Northumberland Strait, where the sGSL population is concentrated, only formed approximately 7000 years ago [35], and so adaptation to this environment appears to have happened within this short time frame, which with a generation time of approximately 22 years in this species [36], equates to a total of just 318 generations. Furthermore, the sGSL and the Scotian Shelf vary significantly in environmental conditions, and especially, there is a 10°C difference in summer bottom temperature between the Scotian Shelf and the much shallower sGSL Northumberland Strait habitat [26]. Such extreme differences may have fuelled rapid adaptation over thousands of years through epigenetic reprogramming, rather than the much slower changes in allele frequencies by natural selection as predicted by Fisher's fundamental theorem [16]. Alternatively, the epigenetic-induced phenotypic adaptation may be acquired at the start of each generation that is born in the warmer sGSL, after adults have over-wintered in the much deeper Laurentian channel, which has similar winter bottom temperatures to the Scotian Shelf [26]. Here, we investigate the possibility of epigenetic variation by comparing transcriptomes among skates of each population, uncovering the patterns of differential expression of key sets of genes responsible for some of the morphological and ecophysiological differences among sGSL and Scotian Shelf winter skate.

**Table 1.** Differences in biology and ecology among populations of the winter skate (*Leucoraja ocellata*) inhabiting the Scotian Shelf and the Northumberland Strait within the southern Gulf of St Lawrence (sGSL). Adapted from [25,26].

	Scotian Shelf population	sGLS population
morphological trait		
tooth row mean (range)	88 (82–93)	74 (62–85)
precaudal vertebrae mean (range)	27 (25–28)	30 (28–32)
max size (cm)	150	68
ecological trait		
max depth range (m)	37–380	1–34
common summer depth (m)	37–110	<21
common summer temperature (°C)	5–10	11–22
age of environment (years)	tens of millions	6000–7000
stress	normal	increased
life-history trait		
max age	21	11
size 50% maturity (cm)	76–77	40–42
age maturity (years)	8–9	5–6
mean egg case length (mm)/wet mass (g)	80.9/41.8	64.1/12.9

## 2. Methods

### 2.1. Samples

Skate samples were collected in July 2009 from the Northumberland Strait in the sGSL from less than 21 m deep, and from the Scotia Shelf at approximately 160 m during Canadian Department of Fisheries and Oceans (DFO) ground fish surveys. Trawls were run for 15–30 min, and live skate were opportunistically sampled from the catches as soon as they were landed. White muscle and liver tissue sampled from adult skates were stored in *RNAlater* for future RNA extraction.

### 2.2. Complementary DNA library construction and 454 sequencing

We investigated the differences in gene expression between individuals by estimating the relative abundance of RNA sequences which are transcribed from genes. The relative abundance of RNA affects the quantities of particular proteins in an organism, which will effect biological processes ranging from cellular development to behaviour. RNA sequences were reverse transcribed into complementary DNA (cDNA), which was then sequenced in order to characterize the genes that transcribed RNAs, and the relative transcription levels (or gene expression), i.e. the transcriptome. Eurofins Genomics performed all laboratory protocols using their standard cDNA library preparation protocol. Briefly, total RNA was extracted from white muscle and liver tissue from two individuals per population (i.e. a mature male and female) and examined by capillary electrophoresis. Total RNA derived from white muscle and liver tissue was pooled in equal amounts per population. From the RNA pools, poly(A)+ RNA was isolated and used for cDNA synthesis. First-strand cDNA synthesis was primed with an N6 randomized primer. Then, 454 adapters A and B were ligated to the 5' and 3′ ends of the cDNA. The cDNA was finally amplified by PCR (11 cycles). Normalization was carried out by one PCR cycle of denaturation and re-association of the cDNA, resulting in N1-cDNA. Re-associated double-stranded cDNA was separated from the remaining single-stranded cDNA (normalized cDNA) by passing the mixture over a hydroxylapatite column. After hydroxylapatite chromatography, the single-stranded cDNA was amplified with 10 PCR cycles. For 454-titanium sequencing, double-stranded cDNAs in the size range of 500–700 bp were eluted from a preparative agarose gel. Aliquots of the size-fractionated cDNAs were analysed by capillary electrophoresis. cDNAs from both pools were used in equal concentration and volume to prepare uniquely tagged 454 sequencing libraries for each population by the manufacture's standard protocol. Both pools were sequenced using one full plate on the 454-FLX titanium platform.

### 2.3. Transcriptome assembly and quality assessment

Next-generation sequencing is prone to producing error when inferring the nucleotide sequence of a DNA strand, particularly at the ends of sequences. This means that quality control and ‘cleaning’ of datasets must initially be carried out so to avoid distorting real patterns of DNA variation. Once these relatively short sequences are of a suitable quality, they are assembled into much longer contiguous sequence fragments (contigs) based on their shared degree of overlap, which represents their relative position in the genome of an individual. Prior to transcriptome assembly, reads were trimmed using custom scripts at 5′ and 3′ ends removing bases lower than Q20 until one greater than or equal to Q20 was met. After quality trimming, we performed de novo assembly of reads for each species using Trinity (release: r20131110 [37]). Contigs shorter than 200 nucleotides (nt) were excluded from further analysis. To assess the quality of assembly, we estimated both the standard quality measure N50, and the fraction of original reads that can be successfully mapped back to the assembled transcriptome. For this purpose, reads from 454 pyrosequencing were split into smaller fragments less than or equal to 60 nt and greater than or equal to 40 nt, and mapped back to their respective transcriptome reference, using Bowtie v. 1.0.0 [38], parameters: -n 1 -5 5 -3 5. We also compared basic assembly statistics with those of the published transcriptome of the closely related little skate (*Leucoraja erinacea*) [39].

### 2.4. Transcriptome annotation

Once the transcriptome was assembled (i.e. long sequences of DNA had been reconstructed from only those genes expressing RNA), we then inferred the function of these contigs (or transcripts) by comparing the DNA sequence variation with that of other organisms where the function of genes had already been identified/inferred. For transcriptome annotation, transcripts were first translated on each one of the six possible open-reading frames (ORFs; +1, +2, +3, −1, −2, −3; i.e. potential codon positions in the DNA from which the gene can start transcription of RNA), and the longest ORF was kept for further analysis. A minimal cut-off of 80 amino acids (inferred from the number of codons in the DNA sequence) was set to consider an ORF valid. Selected translated ORFs (i.e. DNA translated into amino acid peptide sequences) were matched against the UNIPROT database using the *blastp* tool [40], with an e-value cut-off of 1 × 10^−5^. For those transcripts that failed this initial annotation step, the nucleotide sequences were matched against the UNIPROT database using the *blastx* tool. This performed a search against the protein database using a nucleotide query translated on all possible ORFs, and so offered more flexibility in identifying possible functionality. To maximize the number of annotated transcripts, we then used the *hmmscan* tool from the HMMER suite [41] and scanned the longest ORFs with known protein domains from Pfam [42]. Moreover, we annotated possible signal peptides and transmembrane domains, using the SignalP v. 4.1 [43] and TMHMM v. 2.0 [44] tools. For transcripts that failed again to be annotated, a final search was performed using the *blastn* tool against the NCBI nucleotide database. To collate all data, and associate gene ontology (GO) terms to annotated transcripts, we used the Trinotate tool (http://trinotate.sourceforge.net/), BLAST cut-off: 1 × 10^−5^, Pfam cut-off: DGC). Using custom tools, data from the Trinotate output and *blastn* search were aggregated in a single output and annotated in a GenBank-like format.

### 2.5. Differential expression analysis

We developed an approach named ‘comparative expression analysis on consensus transcriptome (CEACT)’ (electronic supplementary material, figure S1) for the comparison and differential expression analysis of transcriptomes. Our approach allows the comparison of transcripts from different populations or species with a sufficiently high degree of identity. First, a conversion table between sGSL and Scotian Shelf skate transcripts was created using the *blastn* tool, with an e-value cut-off of 1 × 10^−20^. Transcripts lacking significant matches were further excluded from the differential expression analysis. For each one transcript that passed the e-value cut-off, the matching region was extracted from both the query and the target sequences. When more than one conserved region across two transcripts was observed, we selected the highest-scoring window between the two transcripts for comparison. Two consensus transcriptomes were built, one using the matching regions from sGSL transcriptome, and one using the regions from Scotian Shelf transcriptome. Bowtie indexes were built for the sGSL and Scotian Shelf consensus transcriptomes using the *bowtie_build* tools, and reads from 454 pyrosequencing for both species were split into fragments less than or equal to 60 and more than or equal to 40 nt. Then, four rounds of reads mapping were performed: (i) sGSL reads mapped on sGSL consensus transcriptome, (ii) sGSL reads mapped on Scotian Shelf consensus transcriptome, (iii) Scotian Shelf reads mapped on Scotian Shelf consensus transcriptome, and (iv) Scotian Shelf reads mapped on sGSL consensus transcriptome. Reads per kilobase per million reads (RPKM) measures were obtained by averaging the RPKM calculated in rounds 1 and 2 for sGSL transcripts, and in rounds 3 and 4 for Scotian Shelf transcripts. The relative difference in the estimated expression level between homologues transcripts of each species was calculated as ‘fold-changes’ (FCs) in the RPKM value. This essentially reflected the premise that the more a transcript had been sequenced was a direct reflection of the relative amount of expression at the corresponding gene. This was calculated as

$$\text{FC}={\text{log}}^{2}\left(\frac{\text{Scotian Shelf RPKM}}{\text{sGSL RPKM}}\right).$$

Finally, REVIGO [45] was used with default settings to reduce GO annotations of differentially expressed transcripts into broader categories of biological function. GO terms are clustered based on their semantic similarity, i.e. reduces similarly described biological functions of transcripts into a single classification, which allows simple visualization of the functional significance of groups of differentially expressed transcripts.

## 3. Results

### 3.1. Transcriptome assembly

We constructed two population-specific cDNA transcriptome libraries of the sGSL and Scotian Shelf winter skate populations, which were compared to identify differential transcript expression. The total number of reads, their length distributions, as well as the number of contigs produced during de novo assembly, the GC content, N50, and other statistics was comparable for the sGSL and the Scotian Shelf individuals (electronic supplementary material, figure S2*a*,*b* and table S1). Furthermore, the transcriptome statistics was similar to those of the closely related little skate (*L. erinacea*) [39]. The quality of each de novo assembly was assessed using the fraction of original reads per species that successfully mapped back to the respective assembled transcriptome. This resulted in 68.6% re-mapping success in the sGSL transcriptome, and 70.5% in the Scotian Shelf transcriptome. For detailed assembly statistics, see the electronic supplementary material, table S1.

### 3.2. Transcriptome annotation and differential expression

**Figure 2.**
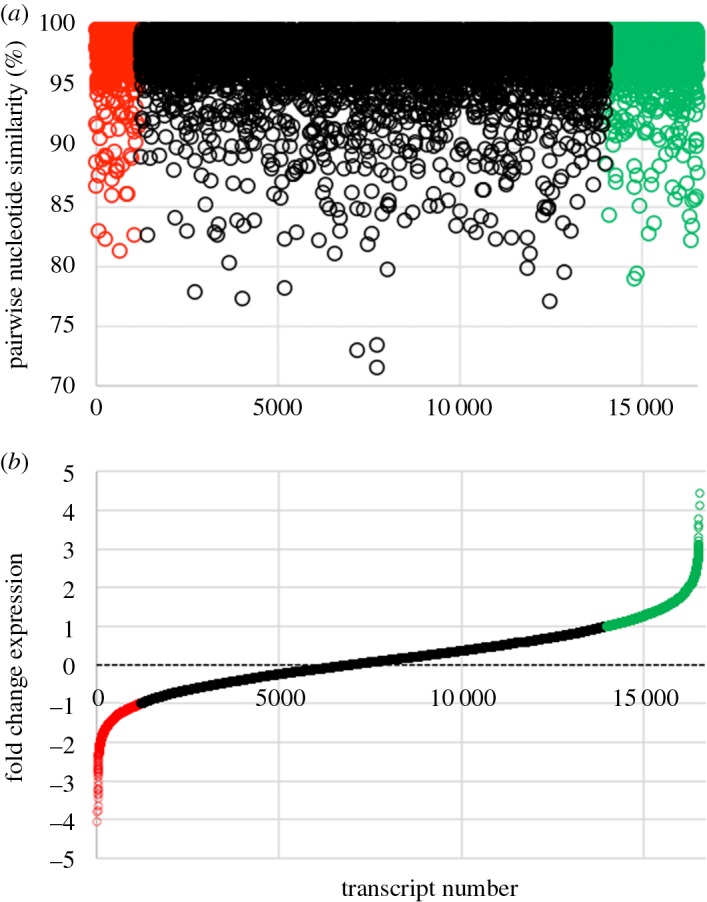
Comparison of (*a*) nucleotide pairwise similarity between 16 519 consensus transcripts of the sGSL and Scotian Shelf winter skate transcriptomes for which RPKM fold-change estimates were made. (*b*) The inferred expression levels of each transcript is represented by the fold-change in RPKM during the re-mapping procedure. A fold change ≥1 (green) or ≤−1 (red) represent transcripts that are either upregulated or downregulated, respectively, in the sGSL population compared with Scotian Shelf population. In total 2475, transcripts were upregulated and 1178 were downregulated in the sGSL skates.

The consensus transcriptome resulted in a final dataset of 16 492 transcripts, of which more than 95% shared at least a 95% sequence identity between the two populations. Among these transcripts, we estimated expression by FC which identified 1178 downregulated transcripts (FC ≥ 1) and 2475 upregulated (FC ≤ −1) transcripts in the sGSL populations relative to the Scotian Shelf population (figure 2 and electronic supplementary material, table S2).

**Figure 3.**
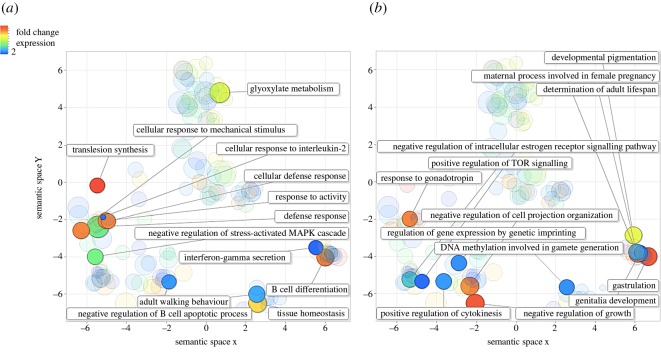
Comparison among clusters of highly upregulated transcripts in the southern Gulf of St Lawrence winter skate population after a reduction in GO term redundancy was applied using REVIGO. Clusters are plotted based on their ‘semantic similarities’ in the GOA database. The colour of each bubble indicates the estimated fold-change difference in transcript expression compared with the Scotian Shelf winter skate, and size indicates the generality in the database where smaller bubbles are less common. For clarity, highly upregulated transcripts are divided by those that are involved in (*a*) immunological processes, responses to stress (e.g. changing environmental conditions), and (*b*) maturation, growth and development.

**Figure 4.**
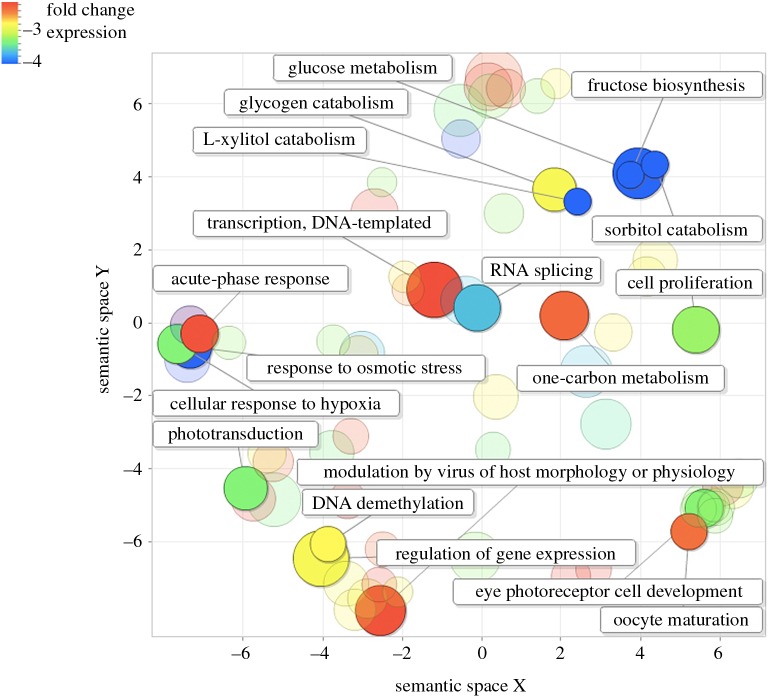
Comparison among clusters of highly downregulated transcripts in the southern Gulf of St Lawrence winter skate population after a reduction in GO term redundancy was applied using REVIGO. Clusters are plotted based on their ‘semantic similarities’ in the GOA database. The colour of each bubble indicates the estimated fold-change difference in transcript expression compared with the Scotian Shelf winter skate, and size indicates the generality in the database where smaller bubbles are less common. For clarity, highly downregulated transcripts are highlighted that are involved immunological processes, responses to stress (e.g. changing environmental conditions), maturation, growth and development, and gene expression.

**Figure 5.**
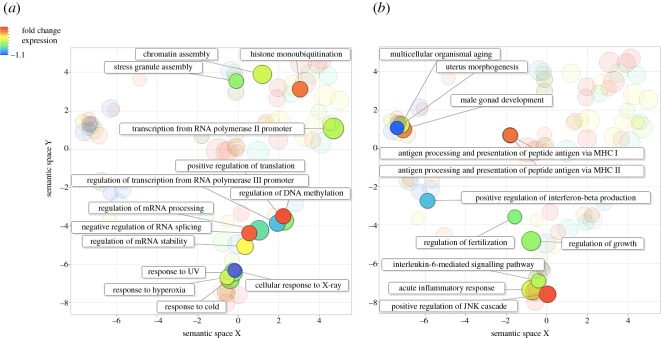
Comparison among clusters of less downregulated transcripts in the southern Gulf of St Lawrence winter skate population after a reduction in GO term redundancy was applied using REVIGO. Clusters are plotted based on their ‘semantic similarities’ in the GOA database. The colour of each bubble indicates the estimated fold-change difference in transcript expression compared with the Scotian Shelf winter skate, and size indicates the generality in the database where smaller bubbles are less common. For clarity, highly upregulated transcripts are divided by those that are involved in (*a*), responses to stress (e.g. changing environmental conditions) and DNA expression, and (*b*) immunological processes, and maturation, growth and development.

Consensus transcripts were annotated and their GO terms were identified. We collapsed GO terms into higher-level categories based on their semantic redundancy and categorized the GO terms of the differentially expressed transcripts. Broadly speaking, two groups emerged. The first group consisted of GO terms that corresponded to known morphological and ecophysiological differences between the sGSL and Scotian Shelf winter skate populations (cf. table 1 and figures 3–5, see Discussion for details). The second group represents GO terms that have previously been associated with expression differences of fishes in response to the effects of warming oceans [46] (electronic supplementary material, tables S3–S5, see Discussion for details)*.* See Data accessibility section for details on raw and processed sequences.

## 4. Discussion

Like many other Elasmobranchs, winter skate (*L. ocellata*) has a life history that is not conducive for rapid evolutionary genetic change [31,32]. Their low fecundity and long maturation times makes them particularly vulnerable to overexploitation and environmental change. Paradoxically, however, skates have persisted over long evolutionary time (approx. 150 to more than 200 Myr [47]), surviving at least two mass extinctions that eradicated more than 75% of the Earth's biodiversity [48]. Furthermore, skates show unusually high levels of endemism in response to varying ecological conditions [33,34]. These observations suggest that resilient species with a modest evolutionary potential such as skate may possess an eco-evolutionary strategy that enables them to persist over macroevolutionary time. Our study suggests that a phenotypically adaptive response through epigenetic regulation may provide this species (and perhaps other elasmobranchs) with a physiological buffer to withstand different environments, enabling it to rapidly respond to novel challenges, which may allow further genetic adaptations to take place over time (see below). We base this on the very different gene expression profiles observed between the two populations, which evolutionary speaking, remain very similar at the DNA nucleotide level owing to only recent separation within approximately the last 7000 years.

### 4.1. Differential gene expression reflects local adaptations and response to climate change

We observed 2475 upregulated transcripts and 1178 downregulated transcripts in the sGSL winter skate compared with the Scotian Shelf skate. Many of the differentially expressed transcripts are linked to GO terms that are consistent with differences in morphology, ecophysiology and life history previously observed between these two populations (see below; table 1 and electronic supplementary material, tables S3–S5) [25,26]. The GO terms of another set of differentially expressed transcripts fit with predictions of physiological changes in fishes responding to the effects of climate change [46,49,50].

Notable changes in gene expression include transcripts involved in exploratory behaviour of new habitats (‘cellular response to mechanical stimulus’) and tissue homeostasis (figure 3*a*). Remarkably, we observed downregulated transcripts associated with winter skate adapting to the warmer and shallower waters of the Northumberland Straight. These included transcripts associated with responses to hypoxia, cold temperatures, ultraviolet and X-rays (figures 4*a* and 5), which correspond to the reduced aerobic capacity in fishes in warmer waters, and also the exposure of this population to significantly higher light levels, compared with the much deep Scotian Shelf. Furthermore, we observed a high degree of downregulation of transcripts involved in eye photoreceptor cell development and phototransduction (figure 4). Both mechanisms are known to be under strong epigenetic control in vertebrates [51,52] and this may represent phenotypic adaptation in response to a reduced requirement for eyes with high light sensitivity in much shallower water.

Intriguingly, we saw altered gene expression patterns among transcripts involved in immune responses. The maternal imprinting of epigenetic regulation of immune genes has been documented [53,54], and in particular in cases where fish were subject to thermal stress [21]. Given that sustained stress can impede immune responses [55,56], the altered expression patterns of multiple immune pathways may represent both the direct effects of this stress, and the epigenetic mechanisms increased to attempt to overcome them. For example, we detected downregulation of transcripts involved in acute inflammatory responses, interleukin-6 and interferon processes, and antigen processing and presentation by major histocompatibility complex (MHC) class I and II (figure 5*b*). Conversely, there was high upregulation of transcripts involved in defence responses of the adaptive and innate immune system, specifically in B-cell differentiation, negative regulation of B-cell apoptosis, interleukin-2 and inferno-gamma secretion (figure 3*a*), which may represent upregulatory mechanisms to compensate for the detrimental effects of thermal stress on other parts of the immune system.

The negative consequences of hypoxia in the sGSL winter skate appear further supported by their differential regulation of transcripts involved in aerobic respiration. In this population, there was downregulation of transcripts associated with glucose, sorbitol, fructose and glycogen metabolism (figure 4), yet upregulation of transcripts associated with glyoxylate metabolism (figure 3*a*), which has been shown to be associated with physiological adaptations to hypoxia [57]. Moreover, we observed the downregulation of transcripts associated with the effects of hyperoxia (i.e. an excess of oxygen), which may be important outside of the sGSL in cooler waters, where individuals are able to deliver oxygen to tissues more efficiently.

This reduced ability for aerobic activity can affect critical life-history traits such as maturation, development, ageing and growth [7,58], and indeed, it is predicted that fish body size will reduce as a response to warming waters [50]. Winter skate in the warmer Northumberland Straight are significantly smaller than those on the Eastern Scotian Shelf (table 1) [25], and remarkably, we observed highly upregulated transcripts which are associated with the negative regulation of growth, and cell projection organization. Similarly, there were upregulated transcripts implicit in the TOR signalling pathway (figure 3*b*), which is critically associated with the regulation of organismal growth in response to stress and hypoxia [59,60]. Moreover, we observed clusters of downregulated transcripts associated with the regulation of growth. We believe that the co-observation of smaller body size, regulation in the expression of genes involved in dealing with poorer oxygen metabolism, and those that regulate growth are a direct reflection of a phenotypic response in winter skate to adapt to the warmer waters of the Northumberland Strait. Although fisheries-induced evolution is also known to reduce body size in fishes [61,62], numerous lines of evidence suggest that this has not been the driver in the adaptive evolution of winter skate body size (see [25] for details), and so supports our observations of epigenetic effects on adaptation to different climates.

Alongside the reduction in body size, winter skate in the Northumberland Strait also have a shorter maturation time, smaller egg cases, shorter lifespan and at least two distinguishing morphological characters compared with the Scotian Shelf population (table 1) [25]. We saw high upregulation of transcripts associated with genitalia and gonad development, negative regulation of oestrogen signalling and pregnancy (figure 3*b*). Similarly, we saw downregulation of other transcripts associated with regulation of fertilization, and uterus and gonad development (figure 5*b*). Many other differentially regulated transcripts associated with development were also observed (electronic supplementary material, tables S3–S5), e.g. gastrulation and developmental pigmentation (figure 3*b*), which may all contribute to the differences observed in morphology and sexual characteristics between the two populations. Notably, multiple transcripts were differentially expressed which are associated with organ development, particularly the heart and kidneys (electronic supplementary material, tables S3–S5). These may both represent adaptive epigenetic responses to stressful conditions such as hypoxia in warmer waters. A previous study observed significant effects of temperature change on reproductive life-history traits in the closely related, little skate (*L. erinacea*) [24], suggestive of temperature-related epigenetic regulation also in this species. Finally, we saw high upregulation of transcripts associated with the determination of adult lifespan (figure 3*b*), and slight downregulation of those associated with ageing (figure 5*b*). Oxidative stress is known to effect organismal ageing by disrupting processes of glucose uptake and lipid synthesis at the cellular level [63,64], and so it is again likely that the differences observed in individual longevity between populations are a result of the effects of hypoxia in warmer waters.

### 4.2. Detection of gene expression regulation

In order for an organism to undergo epigenetic adaptation to a changing environment, there needs to be alteration in gene expression patterns associated with critical biological processes [65]. We observed strong downregulation of transcripts involved in regulation of gene expression, DNA methylation and one-carbon metabolism which directly affect how genes are switched on or off for protein synthesis. By regulating DNA methylation, histone modification and chromatin assembly organisms may differentially express critical genes involved in adapting to warming waters (figures 3–5 and electronic supplementary material, tables S3–S5). Further downregulation of RNA and DNA processes were observed, which are implicit in gene expression and translation from DNA to proteins, and form the underpinning processes required for rapid epigenetic phenotypic adaptation to occur.

Methodologically speaking, although we included four samples of winter skate in transcriptome characterization, by pooling a male and a female per population, we effectively sequenced two biological replicates. For such RNA-Seq experiments, random variation in gene expression patterns may be very small, with highly correlated estimates of sequence fold-change expression between biological replicates (e.g. greater than 0.99 [66]). However, a recent study has shown that increasing biological replicates also increases the power in detecting differentially expressed transcripts in RNA-Seq studies [67]. Therefore, although we are confident in our estimates of differential transcript expression, we also acknowledge that more biological replicates across populations—and additional gene expression detection methods, e.g. bisulfite sequencing to characterize patterns of DNA methylation—would be advantageous for a fuller description of winter skate epigenetic adaptation.

## 5. Implications in the light of climate change

Fossil evidence suggests that elasmobranchs (sharks, skates and rays) originated at least 300 Ma, and underwent rapid divergence in the early Jurassic [68]. Since then, they have withstood many changes in environmental conditions, and numerous mass extinction events. In the study system presented here, the differences in ecology and climate between the sGSL and the Scotian Shelf have only originated within the last 7000 years. The differences in gene expression that we infer to be implicit in adaptation to these relatively new conditions have implications for understanding the success and high degree of endemism of other elasmobranchs, aside from the winter skate. It would be worthwhile to study the effects of climate change on elasmobranches epigenetics across many individuals and multiple populations both experimentally in the laboratory, and in regions that differ markedly in climatic conditions across a species range.

The value of studying epigenetic variation and adaption in fish species that occupy environmental clines may not be limited to elasmobranchs. For example, the characteristic smaller body size of the sGSL skate is echoed also in the sGSL windowpane flounder (*Scophthalmus aquosus*), which occupies a similar oceanographic range [69]. Owing to the significant ecological differences between the sGSL and nearby regions, and its relatively young geological age, the sGSL may represent a valuable ecological system by which inferences can be made on how future rapid changes of environmental conditions may affect adaption of marine biodiversity.

## 6. Conclusion

In summary, our study shows that the winter skate has been able to adapt to a dramatically different environment over short evolutionary time (7000 thousand years), and with apparently little genetic change. Nevertheless, adaptive evolutionary changes in life history, physiology and phenotype have occurred through epigenetic regulation causing changes in gene expression, enabling this species to rapidly respond to novel environmental challenges. Because of its young age and high summer temperatures, sGSL may provide an important natural system to test predictions of climate change on marine biodiversity.

## Supplementary Material

Supplementary Material Figure S1-2, Table S1.doc

## Supplementary Material

Supplementary Table S2.xlsx

## Supplementary Material

Supplementary Tables S3-S5.xlsx

## Data accessibility

Raw 454 sequences reads and quality values can be found in the NCBI database (www.ncbi.nlm.nih.gov/) under accession SRP083109. Assembled transcriptomes for the Scotian Shelf, and southern Gulf of St Lawrence winter skate populations can be found under accession GEZH00000000. All other data required to replicate this analysis can be found on Dryad Digital Repository under accession: http://dx.doi.org/10.5061/dryad.6mr8n [70].
