# Supplementary material for: Adaptive phenotypic response to climate enabled by epigenetics in a K-strategy species, the fish Leucoraja ocellata (Rajidae)
Source: R Soc Open Sci. 2016 Oct 26;3(10):160299. doi: 10.1098/rsos.160299 (PMC5098971; doi:10.1098/rsos.160299)
Supplement: Supplementary Material Figure S1-2, Table S1.doc [file rsos160299supp1.docx]

**Supplementary Information for

Adaptive phenotypic response to climate enabled by epigenetics in a K-strategy species, the fish *Leucoraja ocellata* (Rajidae)**

Jackie Lighten^1*^, Danny Incarnato^2,3^, Ben J. Ward^4^, Cock van Oosterhout^1^, Ian Bradbury^5^, Mark Hanson^6^ and Paul Bentzen^7^

1: School of Environmental Sciences, University of East Anglia, Norwich Research Park, Norwich, NR4 7TJ, UK

2: Dipartimento di Scienze della Vita e Biologia dei Sistemi, Università di Torino, Via Accademia Albertina 13, 10123, Torino, Italy.

3: Human Genetics Foundation (HuGeF), via Nizza 52, 10126, Torino, Italy.

4: Earlham Institute, Norwich Research Park, Norwich, NR4 7UG, UK

5: Department of Fisheries and Oceans, 80 White Hills Road, St. John’s, Newfoundland, A1C 5X1, Canada

6: Department of Fisheries and Oceans, Gulf Region, 343 Université Avenue, Moncton, New Brunswick, E1C 9B6, Canada

7: Marine Gene Probe Laboratory, Department of Biology, Dalhousie University, Halifax, Nova Scotia, B3H 4R2 Canada

*Email: [Jackielighten@gmail.com](mailto:Jackielighten@gmail.com)

*Keywords: Epigenetics, Climate change, Skate, Fish, Phenotypic adaptation, K-strategy*


 **Figure S1.**  Implementation of the “comparative expression analysis on consensus transcriptome” (CEACT) method using two closely related transcriptomes of the southern Gulf of St. Lawrence population (sGSL), and the Scotian Shelf population of Winter Skate (*Leucoraja ocellata*). Once a consensus transcriptome for each population was constructed, relative differences in transcript expression were estimated though the fold change differences in Reads per kilobase per million reads (RPKM) estimated though the mapping stages (See Methods).

**Figure S2.** The frequency distribution of 454 sequencing read length in nucleotides (nt) after quality control for pooled individuals from (a) the southern Gulf of St. Lawrence (sGSL), and (b) the Scotian Shelf populations. The frequency distribution of assembled contig length from quality controlled reads is comparable between (c) the sGSL, and (d) Scotian Shelf transcriptomes. **(a)**

**(b)**

**(c)**

**(d)**

**Table S1.** Statistcs of *de novo* transcripome assembly for the southern Gulf of St. Lawrnece (sGSL) and Scotian Shelf Winter Skate (*Leucoraja ocellata*) compared to statistics from the published transcriptome of the closely relataed Little Skate (*Leucoraja erinacea*)

|  | **sGSL Winter Skate** | **Scotian Shelf Winter Skate** | **Pooled** | **Little Skate** |
| --- | --- | --- | --- | --- |
| **Child contigs** | 27855 | 32041 | 42293 | **-** |
| **Parent contigs** | 25120 | 28284 | 36365 | **-** |
| **Longest contig** | 14286 | 14293 | 30245 | 22530 |
| **Shortest contig** | 200 | 200 | 200 | 74 |
| **Contigs > 1 kb** | 4754 | 6771 | 9768 | 12555 |
| **Contigs > 10 kb** | 2 | 2 | 2 | 10 |
| **Contigs > 100 kb** | 0 | 0 | 0 | 0 |
| **A %** | 29.06 | 29.31 | 29.14 | 27.54 |
| **C %** | 20.78 | 20.48 | 20.71 | 22.55 |
| **G %** | 21.79 | 21.53 | 21.62 | 22.56 |
| **T %** | 28.37 | 28.68 | 28.53 | 27.35 |
| **GC %** | 42.58 | 42.01 | 42.33 | 45.11 |
| **Total contigs** | 27853 | 32039 |  | 103996 |
|  |  |  |  |  |
| **Stats (all transcripts)** |  |  |  |  |
| **N10 (length)** | 2273 | 2679 | 2980 | **-** |
| **N20 (length)** | 1625 | 1905 | 2162 | **-** |
| **N30 (length)** | 1253 | 1476 | 1675 | **-** |
| **N40 (length)** | 995 | 1164 | 1326 | **-** |
| **N50 (contigs)** | 6995 | 7558 | 9218 | 17985 |
| **N50 (length)** | 806 | 936 | 1041 | 757 |
| **Median length** | 529 | 563 | 561 | 319 |
| **Average length** | 702.04 | 772.45 | 805.67 | 550 |
| **Total assembled bases** | 19555189 | 24750189 | 34074089 | 57210566 |
|  |  |  |  |  |
| **Stats (longest isoform)** |  |  |  |  |
| **N10 (length)** | 2172 | 2531 | 2838 | **-** |
| **N20 (length)** | 1529 | 1813 | 2056 | **-** |
| **N30 (length)** | 1179 | 1405 | 1588 | **-** |
| **N40 (length)** | 946 | 1108 | 1252 | **-** |
| **N50 (length)** | 774 | 894 | 988 | **-** |
| **Median length** | 524 | 557 | 550 | **-** |
| **Average length** | 685.47 | 754.73 | 781.23 | **-** |
| **Total assembled bases** | 17218959 | 21346707 | 28409520 | **-** |
